# Association of Cumulative Smoking Exposure with REM Sleep Alterations in Obstructive Sleep Apnea: A Cross-Sectional Study Supported by Exhaled Carbon Monoxide Measurement

**DOI:** 10.3390/jcm15135301

**Published:** 2026-07-07

**Authors:** Kadir Burak Akgün, Derya Yavuz Demiray

**Affiliations:** 1 Department of Pulmonology, Tayfur Ata Sökmen Faculty of Medicine, Hatay Mustafa Kemal University, 31060 Hatay, Türkiye; 2 Department of Neurology, Tayfur Ata Sökmen Faculty of Medicine, Hatay Mustafa Kemal University, 31060 Hatay, Türkiye; derya.yavuzdemiray@mku.edu.tr

**Keywords:** cigarette smoking, exhaled carbon monoxide, obstructive sleep apnea, rem sleep, sleep architecture

## Abstract

**Objective:** The association of smoking with sleep apnea is often based on subjective data. This study quantified the effects of smoking on sleep architecture using exhaled carbon monoxide (eCO) and polysomnography (PSG). **Methods:** A total of 183 patients with suspected obstructive sleep apnea (OSA) were included in this prospective study. Following full-night PSG, eCO was measured within 10 min. Data were analyzed using the Generalized Linear Model (GLM). **Results:** Although initial unadjusted analyses showed an inverse correlation between eCO levels and central apnea count, GLM revealed that male gender was the only independent predictor for central apnea, negating the effect of eCO. GLM analyses, adjusted for age, gender, BMI, and alcohol and drug use, revealed that cumulative smoking load (pack-years) was independently associated after multivariable adjustment with reduced REM sleep duration (B = −0.345, 95% CI [−0.571; −0.119], *p* = 0.003) and REM sleep percentage (B = −0.099, 95% CI [−0.158; −0.040], *p* = 0.001). Similarly, smoking duration (years) significantly predicted decreased REM sleep duration (B = −0.426, 95% CI [−0.724; −0.128], *p* = 0.005) and REM percentage (B = −0.119, 95% CI [−0.197; −0.041], *p* = 0.003). Formal interaction analyses did not detect a statistically significant interaction with body mass index (BMI) (*p* > 0.05 for all interaction terms). **Conclusions:** In OSA, smoking is independently associated with alterations in REM sleep architecture rather than respiratory events. Cumulative smoking load and smoking duration are independently associated with alterations in REM sleep after adjusting for any other major clinical comorbidities.

## 1. Introduction

The relationship between smoking and obstructive sleep apnea (OSA) is complex. Both are common causes of mortality and morbidity, and their association has been shown to increase the impact of comorbidity [1]. Smoking is thought to both disrupt sleep architecture and potentially increase the severity of OSA by triggering inflammation in the upper airways and altering arousal mechanisms [2]. While the acute stimulant effects of nicotine disrupt sleep onset and arousal stability, chronic smoking may lead to more profound alterations in sleep architecture due to persistent inflammation and cumulative carbon monoxide exposure. Furthermore, an association has been observed between smoking and other sleep disorders such as insomnia, parasomnias, arousals, bruxism, and restless leg syndrome. However, due to the nature of existing study designs, evidence regarding causality is insufficient, and a clear distinction between acute and chronic effects has not been established [3]. Smoking leads to thickening and increased edema in the upper respiratory tract mucosa, as well as reversible activation of hypoxia-inducible factor 1 (HIF-1); although sleep quality is seen to be low in current smokers, no significant difference in sleep architecture has been observed between former smokers and never smokers [4]. Although it is thought that smoking cessation may contribute to OSA treatment, evidence is limited. Additionally, the central nervous system effects of pharmacological agents used in smoking cessation can be confounding factors [5,6].

Exhaled carbon monoxide (eCO) measurement is frequently used in active smoking cessation clinics and is one of the objective methods used to demonstrate smoking intensity [7]. The values obtained from measurement in expiratory air also correlate with the Fagerström Test for Nicotine Dependence (FTND), which indicates nicotine dependence [8]. In lung diseases, endogenous CO production may also increase through the induction of heme oxygenase-1 (HO-1), which has anti-inflammatory, anti-oxidant, and anti-apoptotic properties. Sleep studies conducted using eCO in the literature are quite limited, and the results of these studies are inconsistent. Importantly, many previous studies have relied primarily on self-reported smoking habits, which are susceptible to recall bias and fail to adequately distinguish between the acute and chronic biological load of smoking [9]. In the present study, the acute and cumulative effects of smoking on sleep parameters were investigated as distinct entities. To evaluate acute exposure, eCO was utilized as an objective and quantitative biomarker. Conversely, to assess the cumulative burden, total smoking duration (years) and pack-years were analyzed independently of active smoking status. By employing this comprehensive framework, we observed that increasing cumulative tobacco consumption is strongly associated with specific alterations in Rapid Eye Movement (REM) sleep architecture.

## 2. Materials and Methods

### 2.1. Patient Population

Consecutive adult patients with a preliminary diagnosis of obstructive sleep apnea (OSA) who underwent overnight polysomnography (PSG) at the Sleep Disorders Unit from 1 October 2025 to 15 February 2026 were included in this prospective study. Patients were excluded if they refused to participate, had already undergone Continuous Positive Airway Pressure (CPAP) titration, were on CPAP or Bilevel Positive Airway Pressure (BPAP) therapy for device control, had any concomitant chronic respiratory disease (asthma, bronchiectasis, chronic obstructive pulmonary disease, lung cancer), were under 18 years of age or could not perform the PSG procedure satisfactorily. Informed written consent forms were signed by all participants. This study was approved by the Human Subjects Ethics Board of Hatay Mustafa Kemal University (date: 17 September 2025; decision no: 31) and was conducted in accordance with the Helsinki Declaration of 1975, revised in 2013. This study was not registered in a public registry.

### 2.2. PSG Application

Overnight attended PSG was performed using the Alice 6 system (Philips Respironics, Murrysville, PA, USA). Recordings included electroencephalography, electrooculography, submental and bilateral anterior tibialis electromyography, electrocardiography, nasal airflow (pressure transducer), and oronasal thermal sensor thoracoabdominal respiratory effort pulse oximetry body position monitoring.

Sleep stages and respiratory events were scored manually according to the criteria of the American Academy of Sleep Medicine (AASM) as specified in the AASM Manual for the Scoring of Sleep and Associated Events. Apnea was defined as a ≥90% decrease in airflow lasting ≥10 s. Hypopnea was defined as a ≥30% reduction in airflow lasting ≥10 s associated with ≥3% oxygen desaturation and/or arousal. The apnea–hypopnea index (AHI) was calculated as the number of apneas and hypopneas per hour of sleep. OSA severity was classified as mild (AHI 5–14.9 events/h), moderate (15–29.9 events/h), or severe (≥30 events/h).

PSG parameters that were recorded prospectively included overall AHI; REM-AHI; non-REM AHI; number of obstructive, mixed and central apneas; hypopnea count; oxygen desaturation index; total sleep time (min); sleep efficiency; REM latency calculated both from lights off and from sleep onset (min); REM sleep duration (min and percentage of total sleep time); minimum oxygen saturation (minSpO2); and total duration of oxygen saturation below 89% (T89) (min).

Demographic and clinical variables such as age, sex, body mass index, Epworth Sleepiness Scale score, smoking status (current/former/never, including duration, intensity and pack-years), alcohol consumption, and use of hypnotic or sedative medications were obtained from medical records.

### 2.3. eCO Measurement

As a standardized morning procedure, eCO measurements were performed using the Bedfont piCO™ Smokerlyzer^®^ carboxymeter (Bedfont Scientific Ltd., Maidstone, Kent, UK) within 10 min after the completion of the PSG measurement and the patient’s awakening. To ensure standardized smoking abstinence, a strict smoking ban was enforced upon admission to the sleep laboratory at 20:00. Adherence to this smoking ban was objectively verified by the sleep technician through continuous monitoring via direct video surveillance cameras installed in each room. Following preparation, ‘lights out’ was standardized at approximately 22:00 for all participants. This abstinence was strictly maintained under laboratory supervision throughout the night until the morning eCO measurement was finalized. Patients were requested to perform an expiratory maneuver followed by a deep inspiratory maneuver, guided by animation images on the device’s color screen, and to hold their breath for 15 s. Subsequently, they were asked to exhale into the mouthpiece attached to the device upon the animation command. The values appearing on the device screen in ppm and Carboxyhemoglobin (COHb) % were recorded in the dataset.

### 2.4. Power Analysis

Power analysis was performed using G*Power 3.1.9.7 software. Based on the study by Azuma et al. [10], the correlation of r = +0.27 between eCO and T90 was utilized in the calculation. In the model established under the assumption of multiple linear regression analysis (F-test; linear multiple regression: fixed model, R^2^ increase), the following parameters were used: Type I error rate (α) of 0.05, target statistical power of 90%, and a small-to-medium effect size (Cohen’s f^2^ = 0.06). When 1 test parameter and 5 covariates were included in the model, the minimum sample size required was calculated as 178 patients.

### 2.5. Statistical Analysis

Statistical calculations were performed using IBM SPSS Statistics for Windows, Version 25.0 (IBM Corp., Armonk, NY, USA). Gender and smoking status were recorded as categorical variables. Other clinical characteristics, including alcohol consumption, medical comorbidities (Hypertension [HT], Diabetes Mellitus [DM], Coronary Artery Disease [CAD], and psychiatric conditions), and psychotropic medication use (hypnotic, sedative, or antidepressant), were also categorized as present or absent, while other data were recorded as continuous variables. Psychotropic medication use was included as a unified binary categorical variable in the multivariable models, as this variable captured the vast majority of patients with psychiatric conditions and effectively avoided multicollinearity between psychiatric diagnosis and medication use.

The normality of the data distribution was assessed using the Shapiro–Wilk test. Between-group comparisons were conducted using the independent samples *t*-test or Mann–Whitney U test, and the Kruskal–Wallis test was used for three or more groups. Correlation analyses between eCO levels and REM parameters were performed using Pearson’s or Spearman’s correlation coefficients, with 95% CIs calculated via Fisher’s z-transformation.

Multivariable analyses were conducted using Generalized Linear Models (GLMs) to evaluate the independent associations of smoking exposure (cumulative load and duration) with REM sleep parameters. We employed a Gaussian distribution for the dependent variables with an identity link function. The model significance was evaluated using Wald Chi-Square statistics (Type III analysis). To ensure the robustness of our models, multicollinearity was assessed using Variance Inflation Factors (VIFs); all VIF values were found to be within a low range (1.05–1.26), confirming the absence of collinearity among the independent variables (Supplementary Material). Interaction analyses (smoking duration × BMI and pack-years × BMI) were formally tested but excluded from the final models due to non-significance (*p* > 0.05) and the introduction of collinearity. Model diagnostics were verified using Deviance and Pearson Chi-Square statistics. A *p*-value < 0.05 was accepted for statistical significance.

To account for multiple comparisons and to ensure the robustness of our primary findings, a sensitivity analysis was performed using the Bonferroni correction. We adjusted our significance threshold to α*_adj_* = 0.0125 based on the four primary multivariable models assessing the association between smoking exposure metrics (pack-years and smoking duration) and REM sleep parameters (REM duration and REM percentage). Variables showing significance below this adjusted threshold were considered to remain statistically significant after adjustment for multiple comparisons. Findings with *p*-values between 0.0125 and 0.05 were interpreted as secondary or exploratory associations, reflecting the inherent variability in observational clinical data.

### 2.6. AI Statement

The original manuscript draft was prepared in Turkish by the authors. Google Gemini Pro 3.1 was used solely for English translation and language refinement. The authors take full responsibility for all scientific content and the final integrity of the manuscript.

## 3. Results

The mean age of the 183 patients included in the study was 48.92 (±12.33 SD) years, and the male/female ratio was 3.81. The median body mass index (BMI) was 31.60 kg/m^2^, and the median apnea–hypopnea index (AHI) was 30.40. According to their smoking status, the distribution of patients was as follows: 67 active smokers, 17 former smokers, and 99 never smokers. Severe OSA was detected in 51.36% (*n* = 94) of the total population; 12 patients (6.55%) reported sedative/hypnotic drug use; and 34 patients (18.57%) had regular alcohol consumption habits (Table 1).

As expected, eCO was positively correlated with COHb and all smoking consumption parameters (daily amount, years, and pack-years) (*p* < 0.001). While no correlation was found between eCO and BMI (*p* = 0.578) or Epworth score (*p* = 0.239), a negative correlation was observed with age (r = −0.280, *p* < 0.001). No significant correlation was detected between eCO levels and total AHI, REM-AHI, non-REM-AHI, obstructive apnea, or mixed apnea counts (all *p*-values > 0.05). However, a negative correlation was found between eCO values and central apnea count (r = −0.202, *p* = 0.006). No statistical relationship was observed between eCO levels and total sleep duration, sleep efficiency, REM latency, REM duration and ratio, or oxygenation parameters (ODI, T89, minSpO2) (all *p*-values > 0.05). While no relationship was found between eCO and hypnotic/sedative/antidepressant use (*p* = 0.932), it was found to be associated with alcohol consumption (*p* = 0.011, Table 2).

The median eCO value among smokers was 10, with a COHb level of 2.23%; these patients had a median consumption of one pack per day for 20 years. In the comparison based on smoking habits, no statistically significant difference was found between never smokers and former smokers in terms of eCO and COHb levels (adj. *p* = 1.000 for both parameters). In contrast, the eCO values of the active smoker group were significantly higher than both other groups (adj. *p* = 0.008 and adj. *p* = 0.026, respectively). Interestingly, the past cumulative smoking exposure loads (pack-years) of active smokers and former smokers were found to be similar (adj. *p* = 1.000). Pairwise comparisons conducted to determine the source of age differences between groups revealed that the active smoker group was significantly younger than both former smokers (adj. *p* < 0.001) and never smokers (adj. *p* = 0.009). No significant difference in age was observed between never smokers and former smokers (adj. *p* = 0.053).

No differences were observed in AHI, REM-AHI, and non-REM-AHI values according to smoking status (*p* = 0.918, *p* = 0.506, and *p* = 0.821, respectively). Similarly, there was no relationship between obstructive or mixed apneas and smoking status (*p* = 0.783 and *p* = 0.574, respectively). When central apnea counts were examined, it was observed that the number of central apneas in active smokers was significantly lower than in the never smoker (adj. *p* = 0.008) and former smoker (adj. *p* = 0.026) groups. No difference was found between never smokers and former smokers regarding the number of central apneas (adj. *p* = 1.000).

As a result of the Multivariable Generalized Linear Model (GLM) applied to control for potential confounding factors (age, gender, BMI, alcohol use, psychotropic medication use, HT, DM, and CAD), which might have affected the number of central apneas, the relationship between eCO level and central apnea lost its significance (B = −0.679, *p* = 0.138). It was determined that the primary independent predictor of central apnea burden was male gender (B = 16.080, *p* = 0.007) (Table 3).

Similar to eCO levels, no statistically significant relationship was found between REM sleep duration or REM sleep percentage and the number of packs consumed daily (*p* = 0.140 and *p* = 0.111, respectively). In contrast, statistically significant negative correlations were observed between the total years of smoking and total pack-year values—reflecting cumulative smoking exposure—and REM sleep duration (r = −0.163, *p* = 0.028 and r = −0.159, *p* = 0.032, respectively) and REM sleep percentage (r = −0.180, *p* = 0.015 and r = −0.179, *p* = 0.015, respectively) (Table 4).

GLM analyses were performed to control for the effects of potential confounding factors (age, gender, BMI, alcohol, HT, DM, CAD and hypnotic/sedative/antidepressant use) and to identify independent risk factors affecting REM sleep duration (min) and REM percentage (%) within the total sleep time. To evaluate smoking exposure, the variables “Cumulative Load (Pack-Years)” and “Duration of Use (Years)” were included in the models separately (Table 5 and Table 6). Formal interaction analyses (smoking duration × BMI and pack-years × BMI) were also performed to test potential synergistic effects. Both interaction terms yielded non-significant results (*p* = 0.727 and *p* = 0.479, respectively) and introduced severe multicollinearity. Therefore, in accordance with standard statistical principles, these interaction terms were excluded from the final GLMs to allow for an accurate evaluation of the main effects.

In the analyses where REM sleep duration was the dependent variable (Table 5), cumulative smoking load in Model 1 (B = −0.345; 95% CI: −0.571 to −0.119; *p* = 0.003) and smoking duration in Model 2 (B = −0.426; 95% CI: −0.724 to −0.128; *p* = 0.005) were identified as significant predictors after adjusting for all aforementioned confounding factors. Additionally, in Model 2, increasing BMI was also significantly associated with decreased REM sleep duration (B = −0.625, 95% CI: −1.176 to −0.074, *p* = 0.026).

Similar results were obtained in parallel analyses examining the REM percentage within the total sleep time (Table 6). Cumulative smoking load (B = −0.099, 95% CI: −0.158 to −0.040, *p* = 0.001) and duration of smoking (B = −0.119, 95% CI: −0.197 to −0.041, *p* = 0.003) were identified as independent variables negatively affecting the REM percentage. Similarly to the REM duration analysis, it was determined in Model 2 that an increase in BMI was associated with a significant decrease in REM percentage (B = −0.163, 95% CI: −0.307 to −0.019, *p* = 0.026).

While these associations with BMI did not surpass the Bonferroni-adjusted threshold (α*_adj_* = 0.0125), the negative associations of cumulative smoking load and smoking duration with REM sleep parameters remained statistically significant (*p* < 0.0125).

## 4. Discussion

In this prospective cross-sectional study, we investigated the potential effects of smoking on sleep architecture. We differentiated between current smoking status, assessed via eCO levels, and cumulative exposure, quantified through smoking duration and pack-year calculations. Our findings revealed that, while current smoking status showed only weak and gender-dependent associations with central apnea patterns, cumulative smoking exposure exhibited a statistically significant association with alterations in REM sleep architecture. Notably, these associations with REM sleep duration and percentage remained statistically significant even after applying a rigorous sensitivity analysis with Bonferroni correction (α*_adj_* = 0.0125); this suggests that the association between cumulative smoking load and REM sleep was statistically significant even after adjusting for other clinical variables such as BMI, which did not maintain significance under this adjusted threshold.

The relationship between smoking and OSA severity remains contradictory in the literature. Some studies have shown no effect of active smoking or smoking cessation on AHI compared to never smokers [11,12]. In contrast, other studies have reported higher AHI in smokers than in non-smokers [13]. Some researchers argue that AHI is related to the intensity of smoking rather than just the smoking status [14]. In our study, neither smoking habits nor smoking intensity demonstrated any significant effect on AHI. Although a recent meta-analysis indicated that both active smoking and cumulative pack-year consumption correlate with AHI [15], it is well-known that AHI is influenced by confounding factors such as age, gender, alcohol, and BMI. Our findings align with research suggesting that once confounding factors like BMI and age are accounted for, the direct impact of smoking on AHI may diminish, emphasizing that the link between smoking and sleep disorders must be addressed through a multidimensional approach [16].

Evidence suggests that nicotine may reduce apneas through its stimulant effect [17]. Furthermore, decreased CO levels in the carotid body have been shown to increase apneas [18]. In our study, the decrease in central apnea count alongside an increase in eCO levels pointed toward the possibility that nicotine and exogenous CO in cigarette smoke might reduce apneas via their stimulant effects on the central nervous system. However, this relationship lost its significance in multivariable regression analysis, and a high central apnea count only remained associated with male gender. The literature consistently shows that central apneas are more prevalent in men than in women. It has been demonstrated that this difference is attributed to distinct body fat deposition patterns in males, the increased frequency of cardiac issues, reduced chemoreceptor sensitivity relative to women, and hormonal changes, most notably testosterone [19,20,21]. Furthermore, when AHI is evaluated according to sleep stages, it is determined that non-REM-AHI is higher in men, while REM-AHI is not gender-dependent [22]. Consistent with the literature, our results showed that AHI, non-REM-AHI, obstructive apnea count, mixed apnea count, and central apnea count were higher in males independent of other covariates, whereas no significant gender difference was found in REM-AHI. These findings suggest that hormonal characteristics in men may be more dominant and decisive than the effects of stimulant substances in cigarettes.

Studies in the literature investigating the relationship between exhaled carbon monoxide (eCO) levels and sleep disorders appear to be quite limited. It has been suggested that fractional eCO (FeCO) values have high discriminatory power (AUC: 0.824, sensitivity: 0.857, specificity: 0.835) in the diagnosis of moderate-to-severe OSA and can be used as a diagnostic index; however, it should be noted that this high diagnostic accuracy was obtained in a controlled and limited population where primary confounding factors, such as smoking history, were standardized across groups [23]. The lack of a similarly strong relationship between eCO and AHI in our study indicates that the diagnostic sensitivity of this marker may decrease in a broader and more heterogeneous population that includes active smokers and mild cases. The average biological half-life of eCO is approximately 4–5 h, which imposes a strict temporal limit on its utility; furthermore, parameters such as cigarette type, smoking frequency, and inhalation depth significantly influence its concentration. Consequently, eCO is an ideal biomarker for identifying acute smoking exposure rather than cumulative lifetime burden [24]. In another study advocating that eCO is a biomarker directly reflecting the severity of hypoxia, a strong positive correlation was reported between eCO levels and the duration of oxygen saturation below 90% (T90) during sleep. That study hypothesized that intermittent hypoxia increases endogenous CO production by inducing the heme oxygenase-1 (HO-1) enzyme system. However, the fact that the relevant population consisted solely of non-smokers resulted in the exclusion of exogenous eCO load, which is a clinically critical data point, from the analysis. By including active smokers, our study addresses a critical real-world gap that is often overlooked in standardized, non-smoking cohorts [25]. In another study with a methodological approach similar to ours, a weak correlation between eCO and AHI was reported in the general population. Subgroup analyses of that study found that evening eCO levels in non-smoking severe OSA patients were higher compared to mild cases, but this difference weakened and lost its significance in morning measurements. No relationship was observed between eCO and AHI in active smokers [26]. The absence of a significant difference in morning eCO values between mild and severe OSA groups in our study is consistent with both the exogenous eCO effect caused by smoking and the low sensitivity of morning measurements. Furthermore, studies in the literature largely focus on the relationship between the acute effects of smoking and AHI values, while the distinction between obstructive/central apnea or REM sleep parameters has been excluded from the analysis. This situation reinforces the value of our study in addressing existing methodological gaps in the literature.

The characteristics of REM sleep and its relationship with smoking are the most striking parameters examined in our study. REM sleep is critical for memory consolidation, cognitive function, and emotional regulation in humans. Reductions in its duration or percentage have been consistently associated with impaired cognitive performance and compromised emotional stability [27]. Although sleep duration and efficiency decrease with age, REM sleep is generally not affected [26,28]. The effect of alcohol on REM sleep is acute; while it suppresses REM sleep at the beginning of the night, it begins to recover in the following hours [29] and disappears within two days [30]. Although hypnotics and sedatives are thought to suppress REM sleep, a recent meta-analysis revealed that modern hypnotics (lemborexant, daridorexant, zolpidem) do not worsen REM sleep duration or REM-AHI [31]. Regarding the gender–REM relationship, although the REM sleep ratio is lower in men, this effect disappears when analyzed with confounders like age and BMI [32], and large meta-analyses have shown no significant relationship between gender and the REM sleep ratio [28]. In our study, age, gender, alcohol, and drug use showed no effect on REM sleep when analyzed as confounders. The effects of smoking on REM sleep are well-documented; nicotine reduces the REM sleep ratio [33] and shortens its duration [34] when used as replacement therapy. Although an increase in REM sleep percentage after smoking cessation has been suggested, no relationship was found between smoking status and REM sleep characteristics in some studies [12]. Our data show that cumulative exposure, measured in pack-years, is a stronger predictor of REM sleep disturbance than smoking status. In a large cohort with home PSG, no relationship was found between BMI and the REM sleep ratio [35]. Another study showed that REM sleep duration was shorter in obese individuals (BMI > 30 kg/m^2^) compared to non-obese individuals, even when moderate-to-severe OSA patients were excluded [36], which is consistent with our findings regarding the negative association between BMI and REM sleep.

## 5. Limitations and Strengths

Since our cohort consists of patients referred for suspected OSA, it naturally introduces a selection bias. Considering that patients without OSA remained a minority (6.55%), generalizing the results to the general population should be avoided. The inclusion of single-center measurement data is one of the significant limitations of our study. Nevertheless, the required sample size calculated in the power analysis was achieved, thereby ensuring the statistical reliability and robustness of our findings. Although the number of male patients was significantly higher than females, this is an expected situation due to the male predominance in OSA, and the gender effect was mitigated through the use of covariance analyses. Furthermore, the use of the same device for eCO measurement in all patients ensured measurement standardization; however, it should be considered that identical results might not be obtained with different devices. Although patients were instructed to refrain from smoking on the day of the test, compliance with this instruction prior to laboratory admission (at 20:00) relied on self-reporting, and elevated eCO levels in the smoking group suggest that many participants likely did not adhere to this daytime restriction. However, since patients were strictly monitored via continuous video surveillance after entering the sleep laboratory and eCO measurements were taken upon awakening, an objective standardization regarding smoking abstinence was successfully achieved for the critical period immediately preceding the measurement. Furthermore, although our study cohort accounts for major clinical confounders, the potential impact of lifestyle factors (e.g., physical activity, socioeconomic status, dietary habits) on REM sleep cannot be entirely excluded. Therefore, these findings should be interpreted as associations rather than direct biological causality.

## 6. Conclusions

In conclusion, our study indicates that cumulative smoking exposure is independently associated with alterations in REM sleep architecture, even after accounting for BMI and other clinical confounders. While these findings suggest a potential link between chronic tobacco exposure and sleep changes, the cross-sectional nature of our analysis precludes definitive causal inferences. Therefore, we believe that further longitudinal studies—specifically focusing on healthy populations and considering dynamic lifestyle factors—are required to better understand the stability of these associations over time.

## Figures and Tables

**Table 1:** Demographic characteristics and clinical habits of the study population.

Parameters	All Patients (*n* = 183)	Parameters	All Patients (*n* = 183)
Age (years), Mean ± SD	48.92 ± 12.33	BMI, Median (IQR)	31.60 (27.80–37.40)
Gender, *n* (%)		AHI, Median (IQR)	30.40 (13.30–53.50)
Male	145 (79.23%)	eCO (ppm)	
Female	38 (20.76%)	Smokers	10 (6–14)
Smoking Status, *n* (%)		Non-smokers/Former	2 (1–2)
Active smoker	67 (36.61%)	COHb (%), Median (IQR)	
Former smoker	17 (9.28%)	Smokers	2.23 (1.59–2.87)
Never smoker	99 (54.09%)	Non-smokers/Former	0.95 (0.79–0.95)
		OSA Severity, *n* (%)	
		Normal PSG	12 (6.55%)
Sedative/hypnotic use, *n* (%)	12 (6.55%)	Mild OSA	36 (19.67%)
Antidepressant use, *n* (%)	22 (12.02%)	Moderate OSA	41 (22.40%)
Psychiatric illness, *n* (%)	22 (12.02%)	Severe OSA	94 (51.36%)
DM, *n* (%)	36 (19.67%)		
HT, *n* (%)	64 (34.97%)		
CAD, *n* (%)	21 (11.47%)		
Regular alcohol use, *n* (%)	34 (18.57%)		

SD: Standard Deviation, eCO: exhaled carbon monoxide, COHb: Carboxyhemoglobin, BMI: body mass index, AHI: apnea–hypopnea index, PSG: polysomnography, OSA: obstructive sleep apnea, DM: Diabetes Mellitus, HT: Hypertension, CAD: Coronary Artery Disease.

**Table 2:** Correlation between eCO levels and demographic, habit-related, and sleep parameters.

Variables	Correlation Coefficient (r) *	*p*	95% CI
COHb (%)	0.991	<0.001	[0.988; 0.993]
Age (years)	−0.280	<0.001	[−0.408; −0.141]
BMI (kg/m^2^)	−0.041	0.578	[−0.185; 0.105]
Epworth Score	0.088	0.239	[−0.058; 0.232]
Total AHI (events/hour)	−0.062	0.402	[−0.205; 0.084]
Central apnea count (events)	−0.202	0.006	[−0.337; −0.059]
REM sleep duration (min)	−0.054	0.466	[−0.197; 0.091]
REM sleep (%)	−0.084	0.257	[−0.226; 0.061]
Cumulative load (pack-years)	0.593	<0.001	[0.490; 0.679]
Alcohol	-	0.011 **	
Psychotropic drug		0.932 **	

* Spearman correlation coefficient; ** Kruskal–Wallis test was applied. CI: Confidence Interval; COHb, Carboxyhemoglobin; BMI, body mass index; AHI, apnea–hypopnea index; REM, Rapid Eye Movement.

**Table 3:** Multivariable analysis of factors associated with central apnea count (GLM).

Variables	B	Standard Error	*p*	95% CI
Gender (Male)	16.080	5.942	0.007	[4.434; 27.726]
eCO (ppm)	−0.679	0.457	0.138	[−1.576; 0.218]
Psychotropic drug	−13.201	7.032	0.061	[−26.985; 0.583]
Age (years)	−0.033	0.213	0.877	[−0.452; 0.386]
BMI (kg/m^2^)	0.039	0.350	0.911	[−0.648; 0.726]
Alcohol	−4.474	6.336	0.480	[−16.892; 7.945]
HT	−7.490	5.512	0.174	[−18.294; 3.314]
DM	0.816	6.575	0.901	[−12.071; 13.703]
CAD	6.776	7.727	0.381	[−8.369; 21.922]

GLM: Generalized Linear Model, B: Unstandardized Coefficient, CI: Confidence Interval, eCO: exhaled carbon monoxide, BMI: body mass index, DM: Diabetes Mellitus, HT: Hypertension, CAD: Coronary Artery Disease.

**Table 4:** Correlation between smoking habits and REM sleep parameters.

REM Sleep Parameters	Variables	r (95% CI)	*p*-Value
	Daily Cigarette Consumption (packs)	−0.109 (−0.250; 0.036)	0.140
	Smoking Duration (years)	−0.163 (−0.300; −0.019)	0.028
REM Sleep Duration (min)	Cumulative Load (pack-years)	−0.159 (−0.297; −0.015)	0.032
	Daily Cigarette Consumption (packs)	−0.118 (−0.258; 0.027)	0.111
	Smoking Duration (years)	−0.180 (−0.316; −0.036)	0.015
REM Sleep Percentage (%)	Cumulative Load (pack-years)	−0.179 (−0.315; −0.035)	0.015

CI: Confidence Interval, r: Spearman’s correlation coefficient.

**Table 5:** Multivariable analysis results for REM sleep duration (min) (GLM).

Model/Variables	B	SE	*p*	95% CI
**MODEL 1 (CUMULATIVE LOAD)**				
Pack-Years	−0.345	0.115	0.003	[−0.571; −0.119]
BMI (kg/m^2^)	−0.498	0.283	0.078	[−1.052; 0.056]
Age (years)	−0.263	0.168	0.116	[−0.591; 0.065]
Psychotropic Drugs	4.269	5.612	0.447	[−6.731; 15.268]
Alcohol	−2.389	5.014	0.634	[−12.217; 7.438]
Gender (Male)	3.216	4.821	0.505	[−6.232; 12.665]
HT	−3.564	4.398	0.418	[−12.185; 5.057]
DM	5.274	5.339	0.323	[−5.189; 15.737]
CAD	6.696	6.192	0.280	[−5.440; 18.832]
**MODEL 2 (DURATION OF USE)**				
Smoking Duration (Years)	−0.426	0.152	0.005	[−0.724;−0.128]
BMI (kg/m^2^)	−0.625	0.281	0.026	[−1.176;−0.074]
Age (years)	−0.246	0.168	0.143	[−0.576; 0.083]
Psychotropic Drugs	3.488	5.623	0.535	[−7.534; 14.509]
Alcohol	−2.978	5.015	0.553	[−12.807; 6.850]
Gender (Male)	2.666	4.811	0.579	[−6.764; 12.096]
HT	−2.524	4.422	0.568	[−11.191; 6.143]
DM	6.212	5.313	0.242	[−4.202; 16.625]
CAD	7.286	6.266	0.245	[−4.995; 19.567]

GLM: Generalized Linear Model, B: Unstandardized Regression Coefficient, SE: Standard Error, CI: Confidence Interval, BMI: body mass index, DM: Diabetes Mellitus, HT: Hypertension, CAD: Coronary Artery Disease.

**Table 6:** Multivariable analysis results for REM sleep percentage (%) (GLM).

Model/Variables	B	SE	*p*	95% CI
**MODEL 1 (CUMULATIVE LOAD)**				
Pack-Years	−0.099	0.030	0.001	[−0.158; −0.040]
BMI (kg/m^2^)	−0.127	0.074	0.085	[−0.271; 0.018]
Age (years)	−0.031	0.044	0.471	[−0.117; 0.054]
Psychotropic Drugs	0.430	1.462	0.768	[−2.434; 3.295]
Alcohol	−0.806	1.306	0.537	[−3.366; 1.754]
Gender (Male)	0.166	1.236	0.895	[−2.627; 2.295]
HT	−0.927	1.146	0.418	[−3.173; 1.318]
DM	1.199	1.391	0.388	[−1.526; 3.925]
CAD	1.438	1.613	0.373	[−1.723; 4.599]
**MODEL 2 (DURATION OF USE)**				
Smoking Duration (Years)	−0.119	0.040	0.003	[−0.197; −0.041]
BMI (kg/m^2^)	−0.163	0.073	0.026	[−0.307; −0.019]
Age (years)	−0.027	0.044	0.541	[−0.113; 0.059]
Psychotropic Drugs	0.207	1.468	0.888	[−2.670; 3.083]
Alcohol	−0.982	1.309	0.453	[−3.547; 1.584]
Gender (Male)	−0.008	1.256	0.995	[−2.453; 2.469]
HT	−0.635	1.154	0.582	[−2.897; 1.627]
DM	1.482	1.387	0.285	[−1.236; 4.200]
CAD	1.579	1.635	0.334	[−1.627; 4.784]

GLM: Generalized Linear Model, B: Unstandardized Regression Coefficient, SE: Standard Error, CI: Confidence Interval, BMI: body mass index, DM: Diabetes Mellitus, HT: Hypertension, CAD: Coronary Artery Disease.

## Data Availability

The anonymized study dataset and supplementary Variance Inflation Factor (VIF) analyses are publicly available in the Zenodo repository at 10.5281/zenodo.21039202. Researchers are welcome to utilize this dataset, provided that appropriate attribution is given to this study. For any additional information or detailed inquiries regarding the methodology or data, the corresponding author may be contacted directly.

## Supplementary Materials

The following supporting information can be downloaded at: https://www.mdpi.com/article/10.3390/jcm15135301/s1, Table S1: Collinearity diagnostics for REM duration model; Table S2: Collinearity diagnostics for REM percentage model.

## Abbreviations

The following abbreviations are used in this manuscript:

AASM	American Academy of Sleep Medicine
AHI	Apnea–Hypopnea Index
AUC	Area Under the Curve
BMI	Body Mass Index
BPAP	Bilevel Positive Airway Pressure
CAD	Coronary Artery Disease
CI	Confidence Interval
COHb	Carboxyhemoglobin
CPAP	Continuous Positive Airway Pressure
DM	Diabetes Mellitus
eCO	Exhaled Carbon Monoxide
FeCO	Fractional Exhaled Carbon Monoxide
FTND	Fagerström Test for Nicotine Dependence
GLM	Generalized Linear Model
HIF-1	Hypoxia-Inducible Factor 1
HO-1	Heme Oxygenase-1
HT	Hypertension
IQR	Interquartile Range
minSpO2	Minimum Oxygen Saturation
ODI	Oxygen Desaturation Index
OSA	Obstructive Sleep Apnea
ppm	Parts Per Million
PSG	Polysomnography
REM	Rapid Eye Movement
SD	Standard Deviation
SE	Standard Error
T89	Total Duration of Oxygen Saturation Below 89%
T90	Total Duration of Oxygen Saturation Below 90%
