# Supplementary material for: Association of Cumulative Smoking Exposure with REM Sleep Alterations in Obstructive Sleep Apnea: A Cross-Sectional Study Supported by Exhaled Carbon Monoxide Measurement
Source: J Clin Med. 2026 Jul 7;15(13):5301. doi: 10.3390/jcm15135301 (PMC13363570; doi:10.3390/jcm15135301)
Supplement: Supplementary file 1 [file jcm-15-05301-s001.zip › jcm-4407300-supplementary.pdf]

Article Title: Association of Cumulative Smoking Exposure with REM Sleep Alterations in Obstructive Sleep Apnea: A Cross-Sectional Study Supported by Exhaled Carbon Monoxide Measurement

Authors: Kadir Burak Akgün, Derya Yavuz Demiray

Supplementary Tables

Supplementary Table S1. Collinearity diagnostics for REM duration model

Variable	VIF	Tolerance
Psychotropic medication use	1.062	0.941
DM	1.251	0.799
HT	1.261	0.793
CAD	1.081	0.925
BMI	1.101	0.909
Age	1.216	0.823
Gender (Male)	1.057	0.946
Alcohol	1.080	0.926

VIF, Variance Inflation Factor; DM, Diabetes Mellitus; HT, Hypertension; CAD, Coronary Artery Disease; BMI, Body Mass Index; REM, Rapid Eye Movement. VIF values < 5.0 and Tolerance values > 0.2 indicate no evidence of multicollinearity.

Supplementary Table S2. Collinearity diagnostics for REM percentage model

Variable	VIF	Tolerance
Psychotropic medication use	1.062	0.941
DM	1.251	0.799
HT	1.261	0.793
CAD	1.081	0.925
BMI	1.101	0.909
Age	1.216	0.823
Gender (Male)	1.057	0.946
Alcohol	1.080	0.926

VIF, Variance Inflation Factor; DM, Diabetes Mellitus; HT, Hypertension; CAD, Coronary Artery Disease; BMI, Body Mass Index; REM, Rapid Eye Movement. VIF values < 5.0 and Tolerance values > 0.2 indicate no evidence of multicollinearity.
